# Some Observations and Examples of Phlegmasia Dolens, in the Puerperal, Gravid, and Unimpregnated States; and Also in the Male

**Published:** 1819-07-01

**Authors:** 

**Affiliations:** Fellow of the Royal College of Physicians of Edinburgh, and one of the Physicians to the Clifton Dispensary


### Third Department.

### PRACTICAL ESSAYS AND COMMUNICATIONS, *Original or Translated.*

### I.

### *Some Observations and Examples of Phlegmasia Dolens, in the Puerperal, Gravid, and unimpregnated States; and also in the Male.*

By Dr. Dickson, F. R. S. Ed. and L. S.
Fellow of the Royal College of Physicians of Edinburgh, and one of the Physicians to the Clifton Dispensary.

In the following paper I am enabled to adduce some instances of Phlegmasia Dolens in the puerperal, gravid, and unimpregnated female, and also in the male, which have not appeared before the public; and though the observations by which they are accompanied, may not possess much value, they will yet answer a desirable purpose, if they shall draw from the stores of ampler experience than mine, any new facts elucidatory of the history, nature, or treatment of this obscure and distressing complaint. I do not here propose to enter into a minute detail, as the subject has been fully discussed by Mr. White, Dr. Hull, and others; and as the various opinions previously entertained, are to be found in the work of the last-named author.

This disease, under the title of "œdema puerperarum," has been appropriately defined by Dr. Callisen, "tumor elasticus, albescens, renitens, calidus, dolens, foveam impressi digiti haud retinens, puerperis haud infrequentur, gravidis rarissime infestus." Vide Principia systematis Chirur. Hod.

Phlegmasia dolens, indeed, is readily discriminated by the puerperal period of attack, by the unyielding, white, glossy swelling, and by the excessive pain experienced on moving the affected limb. It usually appears in about a fortnight after delivery (though occasionally at both an earlier and a later period) with a feeling of pain, uneasiness, or stiffness in the lumbar, hypogastric, or inguinal region, or upper part of the thigh, with a degree of fullness, or swelling, which soon increases, and extends down the inside of the thigh, and generally to the labium pudendi of the side affected. Sometimes, however, the pain is first complained of in the foot, calf of the leg, or ham; but in either case, the affection quickly diffuses itself; the limb becomes hot, tense, swollen, and of a shining whiteness; and the disease is, for the most part, attended with considerable quickness of pulse, heat of skin, white tongue, anorexy, derangement of secretions, and other symptoms of fever.

The progress and degree of tumefaction vary much; in some cases, the extremity enlarges rapidly, and even in twenty-four hours attains twice the size of the natural state, and sometimes it is larger than this: but in others again, it is much less considerable. The swelling is of a peculiar character; there is no appearance of redness or inflammation—on the contrary, it is pale and glabrous, but warm and incompressible; and the limb feels extremely tender when touched, and exquisitely painful on any attempt to move it, or to let it hang down. After some days, the fever usually abates; the tumour, hardness, and morbid sensibility of the limb become less; and little subcutaneous knobs or prominences are often perceived on drawing the hand over its surface. Sometimes the conglobate glands, and as the intumescence further subsides, enlarged lymphatic vessels are stated to have been felt. Occasionally, as the affection declines in one limb, it migrates to the other. As to duration, the disease occupies a very uncertain period, according to its intensity, the habit of the patient, and the mode of treatment employed. In unfavourable cases, it proves very tedious and untractable, continuing for many weeks, and even months, and very often leaving the extremity for a long time, if not permanently, weakened and enlarged.

In two attacks, in successive confinements of the same lady, where I had a particular opportunity of watching the disease, (one of which occurred on the eleventh day after delivery, the other a day or two later) the invasion was obscure; and a degree of lameness on attempting to walk, imputed to rheumatism, was the first symptom complained of. The last time, slight feverishness, and a diminution of appetite, were experienced on the preceding day; and on inquiry being made, the patient recollected having felt some chilliness, followed by increase of heat in the night; but these were so inconsiderable that they would have escaped notice, if attention had not been excited by the recollection of her former attack, and by the increasing lameness of the limb, almost amounting to paralysis, with some stiffness, and tenderness in the groin; after which the disease rapidly developed itself. On the same evening, the pulse rose from 80, the usual standard, to 100, and afterwards to 112, with some affection of the head, increase of heat, white tongue, quicker respiration, general irritability and restlessness, and the limb became enlarged and hot, exceedingly tender to the touch, and excruciatingly painful on moving, or attempting to put the foot down.

The march of the disease differed in the two instances: in the first, the pain was experienced first in the thigh, or rather in the right groin, from which it extended down the inside of the thigh, in the course of the vessels to the ham, calf of the leg, and foot, in regular succession; in the other, though some flying pains, resembling rheumatism, were felt in the limb, top of the thigh, hypogastric, and inguinal region, yet the pain was chiefly seated, at first, in the calf of the leg, from which it successively descended to the foot, and afterwards extended to the knee and thigh of the left extremity. It is worthy of remark, that on the last occasion, the uterine discharge, (which had become pale, and, indeed, had almost ceased entirely) soon after the accession of fever, returned quite fresh and florid; and continued so, though moderate in quantity, for nearly a week—a circumstance which, with the assistance of leeches, probably prevented the swelling from becoming more considerable, for it did not exceed from half an inch to an inch and a half, or two inches, in any part, the natural dimensions of the limb.

An extraordinary instance of death from uterine hæmorrhage, supervening in phlegmasia dolens, I may here mention, lately came to my knowledge; but as I am not in possession of all the circumstances of the case, I shall only remark, that it appears to me to illustrate strongly the importance of obviating local determination, in such cases, by venesection, when indicated, but particularly the propriety of the abstraction being made at an early period, whether the blood be taken from the system, or locally. A woman, on the fourth day after delivery, was seized with rigors, pain in the foot and leg, which afterwards extended to the thigh and groin. With the intermediate symptoms, and treatment, I am unacquainted; but on the tenth day of the attack, a practitioner was sent for, who directed twenty leeches to be applied to the affected limb, which was nearly twice the size of the other. The bleeding continued after their removal for the greater part of the night; and on her attempting to get up for some purpose, a flow of blood suddenly escaped from the uterus, which proved fatal.

It is altogether unnecessary to advert to the milky deposits and different hypotheses of earlier writers, or to dwell upon the well known opinions of Mr. White, Mr. Trye, Dr. Ferriar, and others, relative to the nature of this disease. They are fully discussed, and the theory that it depends on inflammation and obstruction of the lymphatics of the limb, is combated in the comprehensive Essay of Dr. Hull, who conceives, that "the proximate cause consists in an inflammatory affection, producing suddenly a considerable effusion of serum and coagulating lymph, from the exhalents into the cellular membrane of the limb," (p. 204) which, in the valuable Dictionaries of Dr. Rees and Dr. Parr, is considered, upon the whole, as the most rational explication, and as approaching nearest the truth. By some writers, as Kirkland, Mr. Charles Bell, &c. it has been deemed more of the nature of a critical swelling, as is noticed by the same author, loco citato.

Mr. Burns says, "the disease seems to consist partly in inflammation and partly in nervous irritation, producing both pain and a temporary species of palsy; and the cure consists in lessening the one and allaying the other. I am inclined to consider the cause to be an irritated or slightly inflamed state of the parts within the pelvis, which sometimes produces merely a stiffness and swelling at the passage of the round ligaments, and sometimes an irritation of the nerves which pass to the leg." *Syst. of Midwifery*, 4th Ed. p. 438.

In a Letter, with which I have been favoured by Mr. Charles M. Clarke, he justly characterizes it as a very distressing and obscure complaint; the nature of which is still very imperfectly understood.

The Editors of the London Medical Repository, for June, 1817, observed, that as far as their experience goes, it attacks only those of a rheumatic diathesis; and if they can depend upon a single dissection, that they might venture to pronounce the disease rheumatic inflammation of the coats of the veins of the legs; hence the intermittent character of the fever, and pain, the peculiar state of the urine on the decline of the paroxysm, and the great relief which is experienced from the application of leeches. Dr. John Hall, of Berwick, whose practice in this branch of the profession has been considerable, informs me, that he has met with only three cases of phlegmasia dolens; but that they were not subject to rheumatism. Two of these were much inclined to corpulency, with that pale cast of countenance rather indicative of visceral disease. The bowels were in a very irregular state, and upon using calomel, scyballous stools were produced. The disease, he observes, did not appear to depend upon any circumstance connected with the labour, but upon a certain state of the uterine vessels, or more properly lymphatics, which seems nearly similar to the opinion of Dr. Denman, who believes this disease first to arise in the inguinal glands, by the absorption of some irritating principle in the discharge, the consequence of an unhealthy secretion from the uterus. This idea appears to me extremely plausible in some cases, but it is inapplicable in others. In the case first noticed, and in others I am acquainted with, the patients had been subject to rheumatism; and therefore I was particularly struck with the above remark in the Repository, but as other practitioners with whom I have communicated, have met with the disease in patients who were not rheumatic, I am obliged to conclude, that though such a habit may be more obnoxious to an attack of phlegmasia dolens, it is not confined to those who are of a rheumatic diathesis: and with reference to Dr. Hall's remark, where I have seen the disease, the bowels had been kept freely open subsequent to delivery. Mr. Good thinks the disease in question, which he denominates "Spurganosis puerperarum,"

depends on the tendency to form albuminous secretions at this particular period; but this ingenious idea is not applicable to those instances which have happened, altogether unconnected with the puerperal condition, though it is easy to conceive, that from such a cause, and the greater susceptibility of the system at that time, it should be then more likely to occur. His words are, "the fever seems to be idiopathic, and the effused lymph peculiarly thick and glutinous, constituting a critical deposit; its increased glutinosity resulting, perhaps from the tendency which the general frame at this time possesses, of producing genuine milk, and milk-like or albuminous secretions, in various parts of the body; as, for example, in the peritoneum during typhus puerperarum, and in different parts of the hypogastrium, as described by Puzos, in his *Traite des Accouchemens*, under the name of Depôt Laiteux dans l'Hypogastre.

"That sparganosis is not dependent upon the state of the mammary organ, is obvious, from its occurring in cases where there is no suppression of milk; and from its being found equally in the weak and the strong, the lean and the corpulent, the sedentary and the active, the young and the middle-aged, those who suckle, and those who do not. The œdematous affection that takes place in the ancles afterwards is a mere sequel of the disease, dependent upon vascular debility; and hence Dr. Cullen seems not less improperly to regard sparganosis as a species of anasarca, (anasarca serosa) than others who have regarded it as a species of milk affection. The author has, at the time of writing, a lady under his care, severely affected with sparganosis, of good natural constitution, and with a profuse flow of milk; her age 22; has lain in three weeks." *Syst. of Nosology*, p. 190.

Although, however, this disease does appear to occur under all circumstances of parturition, age, habit, season, and condition of life, and it must be confessed, that the circumstances which influence the effusion of lymph have not been satisfactorily explained, yet those females who have had pain and swelling of the legs during pregnancy, and those who do not give suck, would seem most liable to be afflicted with it; for, of fourteen cases adduced by Mr. White, eight did not nourish their infants, which is a large proportion, when it is considered how much the number of those who suckle, preponderates over those who do not nurse their progeny. Mr. Burns mentions, that if phlegmasia dolens be late of occurring, it is generally in those who have suffered from mammary abscess. The lady already alluded to, who experienced this complaint after her third and fourth confinements, was affected with mammary abscess after the first, and has never been able to nurse with that breast, owing to a defect of the nipple. All her labours, since the first, have been remarkably quick and easy, but her recovery slow.

Upon a review of these later opinions, as to the nature of the swelled legs of lying in women, it appears to me, that none can be admitted unconditionally, and to the total exclusion of the others; for it is probable, that the states which they suppose may be present, in a greater or less degree, in different cases; and, consequently, the above theories may approximate the truth in some instances, and recede from it in others, according to the habit, the date of the attack, the degree and period of the disease, and other peculiar circumstances.

Dr. Callisen says, "*Sedem* huic œdemati præbent extremitates inferiores, una vel utraque; dextram sinistra sæpius invadit morbus; rarissime superiores petit." See his or Dr. Hull's Work, p. 57.

By some authors, it is stated, that "it never attacks either of the arms, or other parts of the body; and though it sometimes occurs in both the lower extremities, in the same, or in different lyings-in, it never attacks the same limb more than once." Vide *Rees's Cyclopædia.*

In the Dictionary of Dr. Parr, who follows that of Motherby, in preferring the denomination of "Ecchymoma Lymphatica," it is observed, "it seldom happens after a miscarriage, nor to a woman more than once."

Many practitioners, however, like myself, have met with it twice in the same individual; and that it never attacks the same limb oftener than once, I entertain some doubt: though it may not be easy to ascertain this point, both from the comparative infrequency of the disorder, and because it often leaves the extremity in an enlarged and weakened state, rendering it difficult to discriminate, on a future occasion, between a second attack if mild, and a mere aggravation of pain and lameness, to which, from occasional causes, its previous morbid condition leaves it obnoxious.

A surgeon of extensive experience in parturient cases, has informed me, that he had met with what he had considered as a second attack of phlegmasia dolens, in the same limb; but on my suggesting the doubts entertained upon this head, as many years had elapsed, he wished to refer me to the subject of the disease for the particulars.

On visiting the lady, however, to whom he obligingly introduced me, I found that her recollection of the circumstances was not sufficiently distinct to prove satisfactory; for she stated, that her leg had never perfectly recovered since the attack which followed her first confinement, about fourteen years before; and that it was still enlarged, and became painful at certain times, particularly on ascending, or taking more exercise than usual. But she could not remember if she had suffered a distinct second attack, after any subsequent accouchement; at least, she had experienced none equal to the first in severity. Perhaps some of my readers may be able to solve this question.

Phlegmasia dolens is by no means a frequent disorder; but neither, on the other hand, is it so rare an occurrence as some authors have represented, nor does such a statement correspond with the "haud infrequenter" in the definition of Callisen already quoted: but it is probably more frequently encountered in some countries and situations than others.

Most of the medical men with whom I have conversed, have met with the swelled leg occasionally, and some of them repeatedly, in the course of their practice.

The following is an extract of a Letter, which I have just received from Dr. M'Arthur, of Walmer, upon this subject:—"I have lately attended a most severe case of phlegmasia dolens, in which the other limb also became slightly, but certainly affected. The lady had suffered severely from cramps in the left hip and extremity for two months previous to her confinement, so much so, that she was never off the bed or sofa, nor had one quiet night until her delivery. The disease appeared very insidiously about a fortnight afterwards, with pain, resembling rheumatism, in the calf of the leg, extending along the course of the muscles. There was no fullness, or at least evident swelling; and she was induced to employ frictions, with some stimulating liniment, which soon decided the nature of the disorder. Leeches and cold applications lessened the disposition to the effusion of lymph; afterwards, tepid oil had a very soothing effect. The bowels were kept open, and occasionally briskly purged. On one occasion, spontaneous vomiting gave great relief. The fever was severe, and had much the appearance of hectic. She is now able to walk about the room; but both ancles swell much in the evening.

At the commencement of the disease, the secretion of milk was much disturbed, and after some time it became necessary to remove the infant from her."

Phlegmasia dolens has, in many instances, succeeded puerperal fever; but I confess the reasons which Dr. Hull assigns for his opinion that a connection and analogy subsist betwixt the two diseases, to me are not satisfactory; though it appears very probable that the peculiarity of circumstances, and of habit, which makes the patient liable to the one complaint, should, also, render her more obnoxious to the other.

I have already instanced uterine hæmorrhage supervening in phlegmasia dolens; and, conversely, we know that the latter disease has, in different instances, succeeded to uterine hæmorrhage. Indeed the excessive loss of blood, from whatever source, sometimes induces a considerable degree of general fever and irritation, which by altering the balance of the circulation and excitability, may dispose to various local complaints, and a change of morbid action, as I recently witnessed in a remarkable instance of intestinal hæmorrhage, which would be foreign to the present detail.

In the Medico-Chirurgical Journal, for May 1818, Mr. Williams, of Portsea, gives two examples, "illustrative of metastasis of morbid action, or increased momentum, from one part of the system to another. It will be seen, that in both cases, there was a transition from the peritoneum to the cellular membrane of the lower extremity, from thence in the first to the cranial contents; and in the second to the mucous membrane of the intestines": and after having noticed a fatal case, which occurred after an easy labour succeeded by alarming flooding, he adds, "it may be worthy of remark, that in the two first instances related above, the attack of phlegmasia dolens was preceded by peritonitis and depletion; and in the third by alarming uterine hæmorrhage." P. 378.

I feel gratified in being permitted to hope, that my former remarks on the utility of blood-letting in fever, may have contributed something to the diffusion of a practice which is now so general. I am too strong an advocate for depletion, and too long confirmed, by an extensive means of observation, in its superior efficacy, to suppose, that any remarks here offered, can be construed to militate against it in fever. But every man of experience must have met with examples where he has, as it were, unchained the fever, by taking away blood. In some cases I have been able to foretel this effect; and in others, where the habit, symptoms, or period of the disease rendered this measure equivocal, I have been puzzled to determine whether it was a signal for the resumption, or interdiction of the lancet. Depletion, as so generally conducting fever to a safer termination, is an invaluable resource conjoined with purgatives; but that it will often not prevent, and *very* often not [or very seldom?] arrest fever, we are too fully assured: whether or not, it sometimes deserves to be considered in the light of a cause, however rarely, becomes an interesting inquiry worthy of attention. *See* Mr. Rumsey's Paper in the last No. of the Edin. Med. and Surgical Journal; and a Case of Epidemic Fever, supervening to Tympanitis, "remarkable from her having been bled repeatedly shortly before the accession of fever, and by having been cupped to the extent of twelve ounces at its very commencement." *Duncan's Clinical Reports, p.* 50.

Phlegmasia dolens, though in a great measure so, is not exclusively peculiar to the puerperal state, or even to the female sex. It has been noticed in a few instances (of which I have seen one) during pregnancy; and in one or two nurses who have lost their children; and also from uterine irritation, altogether unconnected with gestation. I am indebted to the kindness of Mr. Henderson, surgeon in Bristol, for a recent opportunity of witnessing it in a female, aged about 30, in the eighth month of her first pregnancy. Mrs. S. after having walked out on the preceding day, without being sensible of any lameness or uneasiness on going to bed, awoke about 3, A. M. with a pain in the inside and middle of the left thigh, so severe, as to preclude any farther repose. It felt then stiff and swelled, and on examination in the morning, the whole limb was found to be considerably enlarged and "as hard as marble." In the evening she felt feverish, and experienced so much pain that medical assistance was sent for. When I saw her on the fifth day, the pulse was about 100; the hardness was somewhat less, and the pain had been relieved by leeches; but the swelling extended from the groin, including the labium, to the toes; and the limb was about a third larger in circumference than the other, and had the true characteristic appearances of phlegmasia dolens. She noticed a considerable diminution of the renal secretions previously to the attack; but has not been subject to rheumatism.

The same gentleman has favoured me with the following interesting example of its occurrence in the unmarried and unimpregnated female.—"A. B. single, aged 22, of a scrofulous habit, had been in delicate health for three years; but during the preceding six months, had suffered more particularly from hysteralgia. The catamenia were regular, and not deficient in quantity; although the process had of late been attended with much greater pain than heretofore. The pain, however, was not confined to the period of menstruation, but was almost constant in the uterus, and extending from the loins in the direction of the round ligaments. She had also leucorrhœa in an uncommon degree. The treatment consisted of warm bathing, opiates, leeches to the groins and pubis, with some medicines directed to the moderation of the discharge; but these produced scarcely any relief, and that but transient. In this state of things her attention was drawn to a shifting, or rather an extension of the pain down the left thigh, which, in less than thirty-six hours, became the seat of most intense pain, extending from the groin to the very toes.

The pain in the uterine region now decreased very rapidly; and on the third morning of the attack in the leg, she awoke after some very refreshing sleep, free from pain, but with the thigh swoln almost to bursting. She continued ever afterwards free from hysteralgia. The swelling had all the characters of phlegmasia dolens, which I have seen in six instances, including two attacks in the same female. It was pale, shining, tender to the touch, &c.; continued for a week, or ten days, and was cured by blistering and friction. Her health, however, was never re-established; and she has since fallen a sacrifice to pulmonary consumption."—This case is illustrative of the opinion of Dr. Denman, that it may arise from irritation, in consequence of an unhealthy state of the uterus; and of their's, who, with Dr. Ferriar, consider that it may exist (as will be farther shewn) independently of every circumstance regarding parturition. Dr. Thomas, also, in his Practice of Physic, mentions having seen it in an aged woman, and of course unconnected with parturition.

With respect to the existence of phlegmasia dolens in man, Dr. Denmark, in the Medico-Chirurgical Journal for July 1817, describes what he conceives to be a case of it in a male subject, "or a disease in all respects similar;" but the early history and progress of the complaint, previously to its coming under his care, are not sufficien y known to make it easy to give a decided opinion: on dissection the inguinal glands and neighbouring parts were found to be an extensive mass of disease.

In the first volume of the Transactions of the Physico-Medical Society of New York, Dr. Purdy relates an example of phlegmasia dolens which occured in his own person; while in a state of great debility, but recovering from a severe attack of typhus fever, during which he had a painful swelling in the left groin. "At this time, (he observes) my attention was again called to the tumour in the groin; it was considerably enlarged, and extremely painful on pressure, and also when the limb was extended. After remaining in this condition for several days, I was on the 28th of September, 1814, imprudently exposed to a draught of damp, cold air. My left foot soon became unusually cold, and the calf of the leg was affected with pain, similar to that produced by the cramp. The pain continued to increase, notwithstanding the immediate application of warm fomentations, frictions, antispasmodics, &c. and it soon became almost intolerable. After taking large quantities of laudanum, and pressing my foot forcibly against the bed-post, the pain was so much relieved that I fell asleep. *When I awoke in the morning, my leg and thigh were swelled to twice their ordinary size; they had a shining, white appearance, and the skin was smooth, tense, and elastic.* The saphena major and minor veins were much distended; and the whole limb was extremely painful to the touch; or on the least motion. There was an unusual degree of heat in the part, and considerable symptomatic fever. The sensibility gradually diminished for about two weeks; but the swelling continued much the same. At this time, by the constant use of bandages and stimulant applications, its size was so much reduced, and its strength increased, that I was soon able to walk with the assistance of a cane. During the succeeding winter I attended the medical lectures, and, by keeping the limb constantly bandaged, and taking moderate exercise, its strength increased, and I flattered myself that it would soon regain its former vigour. On the 6th of July, 1815, I perceived that my leg was much weaker than usual, and enlarged about an inch in circumference; but by rest, friction with stimulating applications, and the cold bath, it has since regained its former condition of imperfect strength, from which I fear it will never recover." *See* also, the London Med. and Physical Journal, No. 237.

No doubt can be entertained of the above case possessing the true and essential features of phlegmasia dolens; and both in it, and that which preceded, the patients considered exposure to cold as the immediate exciting cause; and it is probable, that a similar effect was produced by the inaction and confined state of the limbs during a long journey, in the case which follows. I have myself seen an instance of the disease appearing the day after imprudently sitting in a damp room, newly washed out; and am inclined to think, that exposure to a damp, cold air, may prove a powerful exciting cause, at a period when the system, debilitated and irritable, is peculiarly susceptible of impressions. This sufficiently shows the propriety of delicate females wearing drawers on first getting up; and guarding against early exposure to cold and moisture after confinement. Mr. Baynton has, also, seen an instance of the disease in question, in an old man from whom, through his influence, I have been obliged with the following account, which I shall give, as nearly as possible, in his own words, only passing over some unessential circumstances. Should the disease not be considered so good a likeness as the former, which, however, it resembles in several respects, it will be recollected that it is not delineated by a medical man; but I am assured by Mr. Baynton, who has seen many cases of this complaint, that it was a well-marked instance of phlegmasia dolens.

Mr. H. B. in returning from Berkshire, in October 1815, was crowded in a remarkably small coach, containing six persons, so as to be unable to move his feet in the smallest degree. On alighting at Buckhampton, he was obliged to hold by a railing for some time before he could walk; such was the lifelessness and numbness of his limbs, after travelling in this manner through the night. Upon arriving in Bath, and feeling the stiffness still remain, particularly under the left knee, he was induced to bathe his feet in warm water. Instead of finding relief, the right leg became as much affected, and the limbs swelled, particularly the right one. Fomenting with flannels, wrung out of a decoction of herbs, produced no good effect; and he was seized with palpitation of the heart, general agitation, and trembling. An apothecary was sent for, who directed cloths dipped in spirit of wine, to be applied to the right limb. In a short time it became more painful, and on the following morning, on attempting to walk a few yards he was obliged to desist, and to raise his leg from the ground. On the next day he was advised to repeat the spirituous application, but the pain and difficulty of walking increasing, he determined at last to send for Mr. Baynton. By this time a week had elapsed since his journey, and he could not now walk across the room, or let the leg hang down any time without violent pain, and visible alteration of the countenance. Upon examination, Mr. Baynton pronounced the case to be a very peculiar one. The appearance was not that of a swelling in the calf of the leg, though greatly enlarged, and the skin bore the resemblance of the gloss of glass. Eighteen leeches were directed to be applied in the afternoon, and the leg bled freely until nine o'clock; after which, linen cloths dipt in equal parts of brandy and water were laid lightly over the limb, and wetted as often as they became dry. He was directed to remain in bed, and during a fortnight the application was continued, and leeches were again twice applied. After this he became so far restored as to bear the application of a bandage; but had not continued so, many days, before he experienced a similar attack in the left leg. The same means were used, with the exception of remaining in bed, and in about a week produced the desired effect; after which time a bandage was applied to that leg also. He still finds it necessary to continue both the rollers; and concludes by stating that he now enjoys good health, and can walk a considerable distance with great ease.

Phlegmasia Dolens then, though, confessedly, extremely rare in any other, is not wholly confined to the puerperal state; and the above instances are not at variance with the researches of Dr. Hull, who in conclusion regards it as, "a complaint, which does not perhaps exclusively affect child-bearing women; but which has rarely, if ever, been observed in males, or even in females differently circumstanced. Indeed the instances are so very few, in which pregnant women, or women who have suckled their infants more than two months, have been observed to be afflicted with this complaint, that it may very properly be regarded as a puerperal disease." P. 212.

Upon the cure of phlegmasia dolens, after what has been said, I shall be brief. So far as I am enabled to judge from my own observation, and that of others, the most successful mode of treatment consists in the free and early use of leeches; in purgatives, cloths wetted with tepid fluids to abstract morbid heat, saline and antimonial medicines according to the degree of fever, quiet and a horrizontal posture, and the compound powder of ipecacuan, or other occasional opiates to allay pain or irritation. Where the patient is of a full, strong habit, with considerable symptomatic fever, it will be proper to take blood from the arm previously, and to enforce the antiphlogistic regimen; but in general, the local abstraction of blood from the limb by means of leeches, assisted by fomentations after their removal, and repeated according to the urgency of the symptoms, will answer the best purpose in the inflammatory stage. If twelve, eighteen, or more leeches be applied at the onset of the disease to the groin, or chief seat of the pain and swelling, and while these are yet confined to the upper part of the thigh, they will often have a powerful influence in arresting its progress, or at least mitigating its future severity; and they should afterwards be repeated in a smaller number, when, and as often as the migratory course of the pain and swelling, indicate their re-application. In both attacks in the individual first mentioned, local bleedings following the track of the inflammation, as pointed out by the pain and tumefaction, though not largely, were repeatedly resorted to, and always with marked relief;—so much so, that the patient wished for, and in one instance, directed the leeches to be re-applied in my absence. Of course in this, as in all other diseases, the depletory measures will be graduated by the symptoms, and peculiar habit of the patient. The application of cloths dipt in tepid water, or goulard, had also a very agreeable and beneficial effect, by abstracting morbid heat; and for this purpose the fluid appeared to answer better when barely tepid, than either of a higher, or lower temperature; and, indeed, such was the excessive sensibility of the limb and of the system, that cold applications could not be borne. After the fever had somewhat subsided and the bowels had been sufficiently acted upon, the Dover's powder, or some other form of opium was exhibited at bed-time with advantage; and when the pain had farther abated, the lactucarium given from five to fifteen grains, or more, appeared to have a tranquillizing effect, by reducing somewhat the frequency of the pulse, and assisting sleep; but it is too mild a sedative to answer this purpose while the pain and irritation are considerable. In some cases of phlegmasia dolens an emetic may be prescribed with advantage; especially if the state of the tongue or stomach should appear to indicate its exhibition. The bowels should be kept freely open in this disease, and occasionally purged by calomel, an infusion of senna, with sulphate of magnesia, &c. The writer on this disease in the Cyclopœdia of Dr. Rees observes, " in three or four cases which we have seen, the importance of free purging appeared to be decidedly established; and while in the first, the use of warm fomentations so far from alleviating the pain in the early stage, seemed to aggravate the sufferings of the patient; in the last treated, both the violence of the pain and the duration of the disease appeared to be greatly diminished, by cooling the limb by frequent *tepid*, and cold washing." I very much concur in this opinion, and am inclined to think, that warm fomentations, even where they do not increase the pain, tend to prolong the disease. As a local application, after the inflammatory symptoms have been somewhat repressed by the above means, frictions with tepid oil may be employed with a soothing and relaxing effect; but unless the complaint be very mild indeed, stimulating liniments in the earlier stages appear to me to endanger the increasing of its duration and severity: at a later period they will prove highly useful. Dr. Porter, of Bristol, speaks in the strongest terms of the efficacy of a blister to the external part of the leg, immediately below the knee, in removing the unwieldiness of the limb, after the febrile symptoms have abated. He relies on moderate general bleeding, and purging during the first stage of the disease, to prevent great distension of the limb; but he says that generally the application of a blister to the exterior part of the leg after the disease has passed its acme, will succeed surprisingly in removing the swelling which remains, and which, in general, proves so tedious and obstinate.

In one of the above cases where I had the advantage of Mr. Clarke's advice, he observed, " In the present instance, as the acute stage must have gone by, I should recommend the application of the camphor liniment twice a-day, with a bandage; a draught of the decoction of bark with ten or fifteen drops of diluted sulphuric acid given twice, or thrice daily, may also assist her recovery."

In the later stage of phlegmasia dolens the continued use of a bandage, by giving great relief and support, proves extremely serviceable; and as the state of the limb then approaches to the nature of œdema, frictions and tonics will be proper, with such other means as are best calculated to promote absorption, and strengthen the constitution.

Clifton, Feb. 1819.

P. S. We may here point out an error in the last edition of Thomson's Dispensatory, page 220. The dose of lactucarium is stated from 1 dr. to 6 oz. instead of from 1 gr. to 6 gr. *Editor.*

